# Sports as a pathway to wellness: sports and health-related quality of life among adolescents in Germany (a cross-sectional analysis from GeWIT Study)

**DOI:** 10.1186/s12889-025-25902-3

**Published:** 2026-01-07

**Authors:** Nessia Rachma Dianti, Anne-Lisa Heye, Michaela Maas, Klaus Weckbecker, Paul Wiesheu, Oxana Klassen, Eva Münster

**Affiliations:** 1 Chair of General Practice I and Interprofessional Care, Institute of General Practice and Primary Care (iamag), Faculty of Health, Witten/Herdecke University, Alfred- Herrhausen-Straße 50, Witten, 58448 Germany; 2 Chair of General Practice II and Patient-Centredness in Primary Care, Institute of General Practice and Primary Care (iamag), Faculty of Health, Witten/Herdecke University, Alfred-Herrhausen-Straße 50, Witten, 58448 Germany

**Keywords:** Sport, Health-related quality of life, Adolescent, Public health, Physical activity, Well-being, Health perception, Youth well-being

## Abstract

**Background:**

Health-Related Quality of Life (HRQoL) comprises individuals’ perceived health status, including physical, psychological and social well-being. Investigating aspects that put adolescents at a good quality of life is important for designing public health efforts to promote their health. This study examined the associations between sports participation and HRQoL among adolescents in Witten, Germany.

**Method:**

This cross-sectional study was conducted from November 2021 to February 2022. Tenth-grade students from nine secondary schools in Witten, who were at least 15 years old, were included (*n* = 649). Data were collected using a self-report written questionnaire. Sports participation was defined as participation in any sports outside of school. Performance-based sport types, such as individual- and team-based sports were also explored. HRQoL (KIDSCREEN-27) was characterized as subjective perceptions of five multidimensional constructs: (1) physical and (2) psychological well-being, (3) parent relations and autonomy (4) social support and peers and (5) school environment. T-values across five dimensions were used to assess HRQoL, with higher values indicating better HRQoL. Descriptive statistics were calculated. Multiple linear regression between sport participation and HRQoL, as well as performance-based sport types and HRQoL adjusted for covariates, were performed. Analyses were adjusted for gender, age, Body Mass Index, subjective socio-economic status, migration background and frequency of physical activity.

**Results:**

Data from 561 participants were included in the analysis. Students who participated in sports showed better physical well-being than adolescents who where inactive in sports (difference (*B*) = 4.92 (*p* < 0.001)). Joining all types of sports was significantly associated with better physical well-being than being sport inactive, but individual- and team-based sports showed the highest increase (difference (*B*) individual-based sports = 3.52 (*p* < 0.001), difference (*B*) team-based sports = 6.14 (*p* < 0.001), difference (*B*) individual- and team-based sports altogether = 9.06 (*p* < 0.001)). Engaging in individual-based sports alone was associated with poorer parent relations and autonomy (difference (*B*) = -2.40, (*p* = 0.048)).

**Conclusion:**

This is the first study to investigate the association between sports participation and HRQoL among adolescents in Witten, Germany. Engagement in sports was positively associated with physical well-being. Participations in individual and team sports outside school could be valuable for adolescents to improve physical activity.

## Background

Health-Related Quality of Life (HRQoL) can generally be defined as self-perceived well-being in the physical, mental, and social domains of health in life [1]. It is usually assessed using several indicators of self-perceived health status as well as physical and emotional functioning [2]. These factors have been suggested to be fundamental health indicators because perceived health and functionality are considered key elements of health status and broader terms of health than clinical measures [3]. HRQoL is related to self-reported chronic diseases and the risk factors, such as physical inactivity, body mass index (BMI) and smoking status [2]. HRQoL measurement can determine the burden of preventable diseases, injuries and disabilities and will help monitor progress to reach the nation’s health goals [2]. Therefore, diagnosing the factors that promote HRQoL is paramount [4].

HRQoL assessment among adolescents has long been a neglected topic compared to that among adults, but it has recently become a focus of health research and increasingly important at the community and national levels for disease prevention and health promotion [5]. Understanding the aspects that contribute to a good quality of life in adolescence is crucial for designing public health strategies and efforts. Considering that adolescence is a crucial time in human development that involves rapid changes in physical, psychological and social well-being.

Prior studies have shown that sports contribute to a more favorable HRQoL among children, adolescents and the general population [6–14]. The studies used varied measurements of HRQoL. In this study, HRQoL based on KIDSCREEN-27 with five dimensions among adolescents is studied. The current study focused on sports participation outside of schools, such as sports club and unorganized sports during leisure hours, the most common settings for being physically active among children and adolescents in Germany [15]. Considering the high interest in sports in our sample, we would like to explore whether sports participation outside the school, which may be less forceful and organized than sports in school, was associated with five dimensions of HRQoL among teenagers in Witten, Germany. To our knowledge, there was no previous study investigating any sports participation outside the school, including sports club and other unorganized sports, and the relationship with HRQoL. We only found one study that investigated sports participation outside the school [16]. However, the study was performed among children. Therefore, the association between sports participation and five dimensions of HRQoL among teenagers in Witten, Germany, is examined.

Most studies on sport participation have emphasized the frequency of being physically active in sport in relation to various HRQoL [6, 7, 9–13]. One systematic review which included 30 studies revealed the varied psychological and social health benefits of sports participation among children and adolescents, with the most commonly cited being enhanced self-esteem and improved social interactions, followed by lower depressive symptoms [17]. Previous studies have shown that better well-being among individuals who participate in sports might be explained by social interaction, and opportunities to build friendships in team sports promote a sense of belonging, which improves mental health [6]. However, studies about this theme, specifically about sport dynamics that look into the accountability of the sport performers through social relationships in teams in relation to HRQoL, especially among adolescents, are lacking. In this current study, individual- and team-based sports were defined as different types of sports to distinguish the sport dynamics and social influence through collaboration within the team. Individual-based sports encompass adolescents’ participation in sports, in which a single person competes individually against others and does not require integration with other individuals to reach a primarily excellent individual goal [18, 19]. Team-based sports were defined as sports in which all group members perform as partners to reach optimal joint scores and have different roles between members and leaders [20, 21]. Little is known about the link between individual- and team-based sports and perceptions health status. Team sports versus individual sports benefits were reported only for individuals’ current health status rather than for individuals’ evaluation of their health and well-being [22, 23].

Findings about individual and team sports and their relation with HRQoL have not been fully elucidated [20, 24, 25]. One study reported that participation in sport clubs, specifically team sports, among children in Australia was associated with more favorable HRQoL than individual sports [25]. In contrast, one study among children in the Netherlands showed that team sports are associated with worse moods and emotions than individual sports [24]. Another study found that individual sports were associated with better overall quality of life than team sports among adult female athletes [26]. In contrast, one study found no significant association between HRQoL and individual versus team sports [20]. Of these studies concerning sport club participation and sport club types, no study has investigated the association between performance-based sports, particularly individual- or team-based sports, and HRQoL among teenagers population. Therefore, this field is explored in the current study.

## Methods

### Study design and setting

This cross-sectional study was conducted from November 2021 until February 2022 in Witten, Germany. It is part of the *Gesunde Stadt Witten* (GeWIT Study) or Healthy City Witten Project. After obtaining ethical clearance (Ethics vote no. 97/2019) from Ethical Committee of Witten/Herdecke University and positive data protection vote (DT-537, DT-630) from data protection officer of the university, consent for the survey was obtained from the responsible education authority of the Ennepe-Ruhr district. After that, school authority was contacted in writing to obtain permission and arrange the data collection. Information letters for students and parents/legal guardians were then sent to the school leadership or designated contact persons from the teaching staff after permission was guaranteed. The information letters were then given two weeks prior to the study.

Data were collected by distributing printed questionnaire during school time. Informed consent, including the study content, objectives, inclusion and exclusion criteria, data protection, voluntary participation, anonymity of the data, and possibility of withdrawing from the study at any time, was discussed on site. Participants completed the self-reported questionnaire by hand during school hour in class. The questionnaire took approximately 25 min to complete. By completing the questionnaire and submitting it, consent was obtained from the participants. Project was funded by German statutory health insurance, The Techniker Krankenkasse.

### Study participants

All 10th-grade students from all secondary schools in Witten, North Rhine-Westphalia, Germany were the target group of this study. The inclusion criteria were 15 years of age or older. Four secondary schools were excluded, including two Waldorf schools who disagreed to participate and two special schools that were excluded due to the specialized approach required for the survey. Ultimately, nine secondary schools agreed to participate and included in the study. Of 757 students from 10th grade, a total of 660 students met the inclusion criteria, after excluding 95 who were absent during class and 2 who were under 15 years of age. Of these 660 students, 7 declined participation and 4 were excluded due to providing unreliable answers, resulting on 649 students agreed to participate in the study and were included in the study. The study received a high response rate of 98.3%. Detailed information about participants’ inclusion and exclusion is presented in Appendix 1.

### Questionnaire instrument/survey

A standardized paper-based questionnaire was used, covering items about well-being and health behavior. The questionnaire was self developed by Institute of General Practice and Primary Care (iamag) Witten/Herdecke University, in which some questions were adapted from previous studies. Questions about sociodemographic and health behavior which were incorporated, were also used in the Robert Koch Institute’s German Health Interview and Examination Survey for Children and Adolescents (KiGGS) as well as the Health Behaviour in School-aged Children (HBSC) [27, 28]. For analysis, items in the questionnaire related to movement and health promotion offers were used to investigate sports participation and items related to well-being were used to measure HRQoL.

### Measures

#### Sports participation

Sport participation is defined broadly to include any sports activities undertaken outside of school subject, including sports clubs and other sports, during leisure hours [29]. It was characterized by two questions in the questionnaire:

1. "Do you join sports?”
  Explanation about the meant sports are all types of sports, including sports club or outside clubs, rather than sports at school was indicated after the question. Options of answers were yes or no. Lists of reasons why not join sports were provided in case “no” was selected.
2. "Which sports do you already join outside the school?”
  Here, students may select either the sports name list, e.g., football, volleyball, or select “I did not join any sport”. Students could also provide any other sports name that they joined in the free-text section.

Answers from both questions were then checked for consistency and any contradictions. Activities that were unreliable and not considered sports such as farming and firefighting were excluded. When the answer about sport participation from one of the two questions was absent (n=26), sport participation was based on one of the provided answers. In this case, twenty-five persons were categorized as being sport-active (n=21) or sport-inactive (n=4). One answer was set to ‘missing’ due to a lack of information on the sport name. In terms of contradictions between the two questions, e.g., sport participation was answered as ‘yes’ in the first question, but ‘I did not join any sport’ was also checked, sports participation was considered missing (n=58). When both answers were not given (n=2) or when provided sports name in the free-text was unreliable (n=2), sport participation was also set to missing. There were a total of sixty-three missing values for sport participation.

#### Individual-based or Team-based Sports 

The type of sport was indicated from the question “Which sports do you already join outside the school?”. A list of various sports names was provided in the questionnaire without a predefined category of individual- or team-based sports. Participants might select the sports name or provide their answers in the free-text field. After all responses on the sports name were recorded, categorization into only individual-based, only team-based, or individual- and team-based sports was done. First, Individual-based sport is defined as a sport in which only one person acts on their own and performs the sport for an optimal individual result [18, 20]. In the questionnaire, cycling, fitness training, jogging, horse riding, swimming, skateboarding and fighting sport were listed and were then classified as individual-based sports in the analysis, following with other sports provided in the free text [23, 30, 31]. Joining only individual-based sports is when participants join individual-based sports exclusively without joining other team-based sports. Second, team-based sport is defined as a sport in which more than one person acts together as a partner, is generally assigned to a team or integrates with others to obtain an optimal joint result [20]. Similar to ‘only individual-based sports’, joining only team-based sports was defined when participants only participated in team-based sports without being active in other individual-based sports. Football, volleyball, and basketball were listed in the questionnaire and were then categorized as team-based sports, together with other sports given by participants in the free text field [22, 23, 30, 31]. Third, both individual- and team-based sports were defined when participants joined individual- and team-based sports at the same time or when the sports can be performed individually or in team (for example, rowing and kayaking). No information on sport type was considered missing (*n* = 4). All sports name provided by the participants can be seen in Appendix 2.

### HRQoL

HRQoL was assessed using the KIDSCREEN-27, a generic self-report tool designed to measure quality of life among children and adolescents aged 8 to 18 years [3, 32]. The KIDSCREEN-27 has been previously shown to be reliable, valid, and sensitive [32]. The scale showed satisfactory internal consistency, with Cronbach’s alphas for the 27-item dimensions ranging from 0.80 to 0.84 [32]. The test-retest reliability is also satisfactory, with Intraclass Correlation Coefficients (ICCs) ranging from 0.61 to 0.74 [32]. The KIDSREEN-27, embedded within KIDSCREEN-52, is a 27-item version instrument involving 5 dimensions of quality of life (QoL) [32–35]. First, *Physical Well-Being* covers items on perceptions of general health, energy and physical activity (five items, e.g., ‘In general, how would you say your health is?’). Second, *Psychological Well-Being* explores items on good moods, life satisfaction, and feeling mentally balanced (seven items, e.g., ‘Thinking about the last week. have you been in a good mood?’). Third, *Parent Relations & Autonomy* covers relationships with parents, the ability to have fun during free time, and financial satisfaction (seven items, e.g., ‘Thinking about the last week, has your father/mother had enough time for you?’). Fourth, *Social Support and Peers* explores relationships with other peers as well as their perceived support (four items, e.g., ‘Thinking about the last week, have you and your friend helped each other?’). Fifth, *School Environment* examines perception about school, ability to learn and concentrate in school, and cognitive capacity (four items, e.g., ‘Thinking about the last week, have you had good ability to pay attention?’) [33]. The items measure the behavior or feelings which are rated using a five-point Likert scale (ranging from “never” to “always” or from “not at all” to “extremely”). In this study, the German language self-reported version of the KIDSCREEN-27 was used, which is already considered a valid, reliable, sensitive and conceptually/linguistically appropriate quality of life measurement in 38 countries [32].

### Potential confounders

Age (category), gender (female, male, and diverse), Body Mass Index or BMI (category), subjective socio-economic status or subjective SES (category), migration background (dichotomous), frequency of daily 60 min Moderate to Vigorous Physical Activity or MVPA (category) were included as covariates in the adjusted analysis. These variables are related to HRQoL, as reported in previous literature [36–43]. All covariate variables were obtained from self-report questionnaires. International standard of BMI unit was used (in kg/m^2^). BMI categories of underweight (≤ 50th percentile, P50), normal weight (≤ 90th percentile, P90), overweight (>P90 to ≤ P97) and obesity (>P97) were defined according to national German percentiles based on Kromeyer-Hauschild [44]. The reference values were based on age and sex [44] and were currently used to define BMI among children and adolescents in Germany in line with the recommendations of the Study Group on Obesity in Childhood and Adolescence (AGA) and the nationwide representative study, KiGGS Study. In the case of diverse gender participants, BMI categorization was based on female percentile.

Subjective SES was recorded as low, medium or high using the German version of the McArthur scale, which was also used in the KiGGS Study from the Robert Koch Institute (RKI) [45]. The variable migration background is defined by the German Federal Statistical Office [46]. Frequency of daily 60 min. MVPA was defined from the question “over the past 7 days, on how many days were you physically active for a total of at least 60 minutes per day?”. The response options included 0 to 7 days. Responses were then categorized into four categories on the basis of the World Health Organization (WHO) recommendation on MVPA at least 60 min per day across the week, which has moderate agreement based on Cohen’s Kappa (0.503) [47, 48]. Adolescents with daily 60 min of MVPA on between 0 and 2 days (inactive), 3–4 days (slightly active), 5–6 days (almost fulfill the recommendation), and 7 days (fulfill the recommendation) were determined [47].

### Data analysis

The data were digitally transformed from the questionnaire using Scan-Systems FormPro. All statistical analyses were conducted using IBM SPSS Statistics (version 28), with a significance threshold set at *p* ≤ 0.05. Before analysis, a plausibility test was conducted and normality of the HRQoL was examined using the p-value from the Kolmogorov-Smirnov test, which p-value >0.05 indicates a normal distribution [49].

Sociodemographic data for all participants, along with stratification by sports participation, are presented as descriptive statistics using frequencies and percentages. BMI values for overweight and obese individuals are presented together because of small values and data privacy protection [50]. Differences in sociodemographic data and participants’ characteristics across the sports participation groups were examined using the p-value Chi-Square Test. The relationship between the independent variable of sports participation (yes/no) and the dependent variables of five dimensions of HRQoL was assessed. The association between performance-based types of sports (including non-participation in sports, individual-based sports, team-based sports, and individual- and team-based sports) and HRQoL was also explored.

HRQoL was evaluated from five dimensions of the KIDSREEN-27 using a standardized T-Value, which indicates HRQoL with higher values indicating better HRQoL [3]. Before summing up all the answers, some of the items were recoded, in which a higher score indicated a more favorable HRQoL [3, 35]. All responses from each dimension were then summed up and assigned to Rasch Person Parameters (PP) [3]. The PPs were transformed into T-Values with a mean of 50 and a standard deviation of 10 [3]. The T-value was calculated only if there was not more than one missing answer to the questions in each dimension [3, 51]. Median and interquartile range of T-values of five dimensions from the whole participants, as well as stratified by sports participations, were reported because of the non-normally distributed data [52–54].

Simple linear regression between sports participation and HRQoL was done, as well as between the types of performance-based sports and HRQoL. Multiple linear regression analysis adjusting for the covariates age, gender, BMI, subjective SES, migration background and frequency of daily 60 min of MVPA was performed. The unstandardized regression coefficient (B) was used to assess differences in HRQoL between groups based on sports participation and performance-based sports types. This coefficient represents the expected change in HRQoL T-values for a given group relative to the reference group. Missing values for sports and HRQoL were excluded. Missing values from covariate variables were treated with Most Common Imputation method and assigned to the group with the highest n, as long as the n was under 5% (age: *n* = 7, migration background: *n* = 9, physical activity: *n* = 9) [55]. When the percentage was above 5%, missing values were set to a new fixed value and assigned to a separate group with a new dummy variable, which indicated missing in the analysis (subjective SES: *n* = 36 and BMI: *n* = 175) [56, 57]. Missing in gender was assigned to the female group (gender: *n* = 1). The findings were considered statistically significant, as the obtained p-value was less than 0.05.

## Results

A total of 649 of the 660 students completed the questionnaire and participated in the GeWIT study. From the 649 adolescents who participated in the study, 561 (86.4%) were included in the analysis. Data from 88 teenagers (13.6%) were excluded in the analysis due to missing data on sport participation (*n* = 61), type of sport (*n* = 4), HRQoL (*n* = 21), and both sport participation and HRQoL (*n* = 2) (see Appendix 1).

Table 1 provides a detailed overview of the participants’ general characteristics. Frequencies of females and males were almost distributed equally (46.9% vs. 48.5% respectively) and there was 4.6% of participants with gender of diverse. More than half of the participants were 15 years old (59.7%), had high SES (53.5%), were of normal weight (53.8%) and no history of migration (56.7%). Around 33% of participants had 60 min. of MVPA in 3–4 days. A high number of 468 adolescents participated in sports (83.4%). Slightly more than half of all participants engaged in individual-based sports (50.8%). Almost all participants reported an interest in sports (92.5%). There were significant differences of all characteristics between teenagers who participated in sports and did not participate in sports, particularly gender (*p* < 0.001), age (*p* = 0.009), subjective SES (*p* < 0.001), BMI (*p* = 0.04), migration background (*p* < 0.001), frequency of daily 60 min MVPA (*p* < 0.001), type of sports (*p* < 0.001) and interest on sports (*p* < 0.001). Those who were significantly more likely to participate in sports than other groups were those who were male, in the age group of 15 years old, had high subjective SES, normal BMI, no migration background, were physically active for 3–4 days a week, performed individual-based sports and had an interest in sports. Teenagers who participated in sports had significantly higher scores of HRQoL in three out of five dimensions, such as physical well-being (p-value < 0.001), psychological well-being (p-value < 0.001) and social support & peers (p-value = 0.003).

**Table 1:** General characteristics and sports participation of the GeWIT-Study participants (*n* = 561)

Participants’ characteristics	Total (*N* = 561)		Sport participation				*P*-Value
			Yes (*n* = 468)		No (*n* = 93)		
	*N*	%	*n*	%	*n*	%	
Gender							< 0.001^b^
Female^a^	263	46.9	207	44.2	56	60.2	
Male	272	48.5	243	51.9	29	31.2	
Diverse	26	4.6	18	3.8	8	8.6	
Age							0.009^b^
15^c^	335	59.7	292	62.4	43	46.2	
16	175	31.2	134	28.6	41	44.1	
≥17	51	9.1	42	9	9	9.7	
Subjective SES							< 0.001^b^
Low	39	7	20	4.3	19	20.4	
Medium	186	33.2	164	35	22	23.7	
High	300	53.5	251	53.6	49	52.7	
missing	36	6.4	33	7.1	3	3.2	
BMI							0.04^b^
Underweight	30	5.3	22	4.7	8	8.6	
Normal weight	302	53.8	259	55.3	43	46.2	
Overweight & obese	54	9.7	49	10.5	5	5.4	
Missing	175	31.2	138	29.5	37	39.8	
Migration background							< 0.001^b^
No^c^	318	56.7	280	59.8	38	40.9	
Yes	243	43.3	188	40.2	55	59.1	
Physical activity frequency							< 0.001^b^
0–2 days	115	20.5	67	14.3	48	51.6	
3–4 days	183	32.6	168	35.9	15	16.1	
5–6 days	154	27.5	135	28.8	19	20.4	
7 days^c^	109	19.4	98	20.9	11	11.8	
Type of sport							< 0.001^b^
Not join sport	93	16.6	0	0	93	100	
Individual-based sports	285	50.8	285	60.9	0	0	
Team-based sports	49	8.7	49	10.5	0	0	
Individual- & team-based sports	134	23.9	134	28.6	0	0	
Interest on sport							< 0.001^b^
No	42	7.5	5	1.1	37	39.8	
Yes	519	92.5	463	98.9	56	60.2	
KIDSCREEN-27 (median, IQR)							
Physical Well-being	42.5	38.2–49.6	44.7	38.5–52.4	36.5	32.7–40.4	< 0.001^d^
Psychological Well-being	43.2	36.6–50.6	44.6	37.9–50.6	40.4	31.9–46.5	< 0.001^d^
Parent Relations & Autonomy	51.2	45.2–55.7	51.2	45.6–55.7	50.1	44-55.7	0.4^d^
Social Support & Peers	46.9	42.1–53.2	46.9	42.1–53.2	44.4	35.8–53.2	0.003^d^
School Environment	45.4	40.7–51.1	45.4	40.7–51.1	45.4	38.7–51.1	0.1^d^

^a^missing in gender (*n* = 1) is included in the female group

^b^P-value Chi-Square Test

^c^missing is included in the group with the largest n for multiple linear regression (n age = 7, n migration background = 9, n physical activity = 9)

^d^*P*-value Mann-Whitney Test

Drop-out analysis showed significant differences in subjective SES (*p* = 0.02), migration background (*p* = 0.03), and no significant differences in other characteristics between teenagers who were included and excluded in the analysis. Adolescents whose information was not used due to missing data showed worse scores of physical well-being (*p* = 0,03), psychological well-being (*p* = 0.04), school environment (*p* = 0.01), and no significant difference of other dimensions was found.

Table 2 presents the associations between sports participation and five dimensions of HRQoL among adolescents. It was shown from the multiple linear regression analysis that joining sports is strongly associated with better physical well-being than being sport inactive (*B* = 4.92, β = 0.18, *p* < 0.001). It was indicated that participating in sports was associated with a 0.18 standard deviation increase in HRQoL T-Value, which indicates a small positive effect size. We did not find a significant association between participating in sports versus being sport inactive and psychological well-being, parent relations & autonomy, social support & peers and school environment.

**Table 2:** Associations between sports participation and HRQoL among the GeWIT Study participants (*n* = 561)

HRQoL dimensions	Sports	Crude Analysis				Adjusted Analysis^a^			
		B^b^	β^c^	*p*-value^d^	95% CI	B^b^	β^c^	*p*-value^e^	95% CI
Physical Well-Being	no	Reference group				Reference group			
	yes	8.79	0.32	**< 0.001**	6.64–10.93	4.92	0.18	**< 0.001**	2.76–7.09
Psychological Well-Being	no	Reference group				Reference group			
	yes	3.99	0.14	**0.001**	1.62–6.37	0.27	0.01	0.83	2.17–2.70
Parent Relations & Autonomy	no	Reference group				Reference group			
	yes	1.43	0.05	0.19	-0.73-3.59	-1.70	-0.06	0.14	-3.95-0.55
Social Support & Peers	no	Reference group				Reference group			
	yes	3.98	0.14	**< 0.001**	1.72–6.24	1.35	0.05	0.28	-1.09-3.79
School Environment	no	Reference group				Reference group			
	yes	1.56	0.07	0.12	-0.40-3.52	-0.35	-0.15	0.74	-2.49-1.78

Significant p values are in bold

^a^adjusted for gender, age, BMI, subjective SES, migration background and frequency of daily 60 min of MVPA

^b^Unstandardized regression coefficient

^c^Standardized regression coefficient

^d^*P*-value simple linear regression

^e^*P*-Value multiple linear regression

Table 3 shows the associations between the types of performance-based sports and five HRQoL dimensions. Participation in individual-based sports alone was associated with better physical well-being than not participating in any sport (*B =* 3.52, β = 0.17, *p* = 0.001). A small positive effect size was shown. However, in comparison with not joining sports, participation in team-based sports showed even more favorable physical well-being than joining only individual sports (*B =* 6.14, β = 0.17, *p* < 0.001). A small positive effect size was also indicated. It was shown that, in comparison with not participating in sports, performing individual- and team-based sports had the most favorable physical well-being, compared to joining only individual-based sports or only team-based sports (*B =* 9.06, β = 0.38, *p* < 0.001). There was a medium positive effect size in joining both individual- and team-based sports. Participating in only individual-based sports was significantly associated with a worse score of parent relations & autonomy than not joining any sport (*B =* -2.34, β=-0.12, *p* = 0.04). A small negative effect size was found in the association. No significant difference in psychological well-being, social support & peers and school environment between types of sports was observed.

**Table 3:** Associations between performing individual or team sports and HRQoL dimensions among the GeWIT Study participants (*n* = 561)

HRQoL dimensions	Sports type	Crude analysis				Adjusted analysis^a^			
		B^b^	β^c^	*p*-value^d^	95% CI	B^b^	β^c^	*p*-value^e^	95% CI
Physical Well-Being	Not join	Reference group				Reference group			
	Individual-based only	6.05	0.30	**< 0.001**	3.93–8.17	3.52	0.17	**0.001**	1.35–5.68
	Team-based sports only	9.87	0.27	**< 0.001**	6.74–13.01	6.14	0.17	**< 0.001**	3.05–9.22
	Both individual- and team-based	14.20	0.60	**< 0.001**	11.81–16.60	9.06	0.38	**< 0.001**	6.52–11.6
Psychological Well-Being	Not join	Reference group				Reference group			
	Individual-based only	1.67	1.23	0.18	-0.76-4.09	-0.83	-0.04	0.51	-3.32-1.66
	Team-based only	6.74	1.82	**< 0.001**	3.15–10.32	2.67	0.07	0.14	-0.88-6.22
	Both individual- and team-based	7.96	1.39	**< 0.001**	5.22–10.69	2.65	0.10	0.07	-0.27-5.57
Parent Relations & Autonomy	Not join	Reference group				Reference group			
	Individual-based only	0.29	0.29	0.79	-1.96-2.55	-2.34	-0.12	**0.04**	-4.66–0.03
	Team-based only	3.92	3.92	**0.02**	0.59–7.25	0.80	0.02	0.63	-2.49-4.10
	Both individual- and team-based	2.93	2.93	**0.02**	0.38–5.48	-0.94	-0.04	0.50	3.65–1.78
Social Support & Peers	Not join	Reference group				Reference group			
	Individual-based only	3.22	0.16	**0.008**	0.85–5.60	0.76	0.04	0.55	-1.75-3.28
	Team-based only	4.34	0.12	**0.016**	0.83–7.85	2.65	0.07	0.15	-0.94-6.24
	Both individual- and team-based	5.46	0.23	**< 0.001**	2.77–8.14	2.61	0.11	0.08	-0.34-5.57
School Environment	Not join	Reference group				Reference group			
	Individual-based only	-0.03	-0.001	0.98	-2.05-2.00	-1.34	-0.08	0.23	-3.52-0.84
	Team-based only	4.54	0.14	**0.003**	1.55–7.54	2.59	0.08	0.10	-0.52-5.69
	Both individual- and team-based	3.84	0.19	**0.001**	1.55–6.13	1.34	0.06	0.30	-1.22-3.90

Significant p values are in bold

^a^adjusted for gender, age, BMI, subjective SES, migration background and frequency of daily 60 min of MVPA

^b^Unstandardized regression coefficient

^c^Standardized regression coefficient

^d^*P*-value simple linear regression

^e^*P*-value multiple linear regression

## Discussion

The primary objective of this study is to investigate the associations between sports participation, categorized as joining sports and not joining sports, and five dimensions of HRQoL among teenagers in secondary school. Furthermore, this research aims to explore the link between performance-based sports types, especifically individual-based versus team-based sports, and HRQoL. It was shown that sports participation is strongly associated with better physical well-being than not joining any sport. No significant association was found between the other four dimensions of HRQoL and sports participation. The explorative analysis of individual- versus team-based sports showed that participating in individual-based sports alone was associated with better physical well-being than being sport inactive. However, compared to the individual-based sports, joining team-based sports showed even more favorable physical well-being than not joining a sport. We have found that participating in both individual- and team-based sports is associated with more favorable physical well-being than not joining any sport and it appears to be the most favorable, compared to joining only individual- or only team-based sports. Regarding parent relations & autonomy, it was shown that participating in individual-based sports was associated with a worse score of parent relations & autonomy than not joining sports. No significant relationship between performance-based types of sports and the other three dimensions of HRQoL, namely psychological well-being, social support and peers and school environment.

The positive association between participating in sports and physical well-being aligns with the findings of previous studies [6, 20, 58–60]. These studies reported better HRQoL among teenagers who became sport club members. An explanation for the more favorable HRQoL in physical well-being among adolescents who are engaged in sports could be that they are more active, which may lead to a healthier lifestyle [61]. A previous study of students who attended sports-related vocational training programs found that those who were more physically active tended to have fewer health limitations and bodily pain than students who were more sedentary [61]. In this sense, students who are sport active may have good physical condition, good ability to perform daily activities and better perceptions of their health status and physical wellbeing [61, 62].

The positive association between individual-based sports and physical well-being in comparison with not joining sports may be associated with the impact of being physically active, as previously mentioned. Additionally, performing individual-based sports may provide the opportunity to concentrate without distraction when practicing alone [63]. It was previously reported that a higher percentage of individual adolescent athletes had the purpose of doing sports as a goal-oriented reason than team sport athletes [63]. Teenagers in individual sports may benefit from better preparation, as they can rely solely on individual skills to achieve their personal goals, for example, reducing body fat or increasing muscle strength [63, 64].

No positive association was found between sports participation and other HRQoL dimensions, except for physical well-being. One study among children has shown that sports club participation was associated with the parents and peers domains, but not with psychological well-being and school environment [20]. It could be that being sports active in adolescent population was not sensitive enough to be linked with other dimensions other than physical aspects. There could be many other factors other than being sports active, which matter to the association with social and psychological aspects. Regarding the school environment, our finding was supported by previous study, which also found that sports club participation outside the school was not significantly associated with school environment in children population, but school-sports club participation was significantly linked with school environment [16]. This could be because the participation in sports outside the school was included in the current study and it may have no clear relationship with school aspects.

While performing individual-based sports was associated with better physical well-being than being sport inactive, joining team-based sports was also associated with even more favorable physical well-being compared with sports inactive teenagers. Organized youth sports, such as team sport, is competitive in nature and involves rules and formal practice [65]. Participating in increasingly competitive sports like football leads to additional pressure to meet specific performance standards. For example, football athletes may be encouraged to increase their weight, lift weights, improve flexibility and perform several activities such as running to be judged as competitive by their trainers or coaches [66]. Those aspect seems to be associated with a ‘healthy feeling’ and may improve the adolescents’ physical well-being in team sports. However, it should be further studied in the future studies.

The higher increase in physical well-being score among adolescents who joined team-based sports than individual-based sports may be explained by cardiorespiratory endurance, coordination, and power and speed [67]. Cardiorespiratory fitness, which has many health benefits and reduces the risk of cardiovascular disease through oxygen supply to the respiratory system, was previously studied to be positively associated with the physical component in HRQoL across varied life-span including among adolescents [68–71]. Team sports support better cardiorespiratory endurance, which was also found to be the highest among athletes aged 11–21 years old in team sports [67]. Cardiorespiratory endurance is better in sports with intermittent aerobic efforts such as football and basketball in comparison with dancing and horse-riding, which have less demand for cardiorespiratory endurance and concentrated more on performance, flexibility, and arm strength, just like in swimming, even though in basketball too [67, 72, 73]. It may also be explained by power and speed, which are physical fitness determinants of team sports and were previously found to be higher among team sports athletes [74]. However, it requires further study. Performing both individual and team sports may be combined into a profound benefit and probably what leads to the highest increase in physical well-being [17, 25]. Only one study showed a positive association between individual and team sports with better HRQoL [25]. However, it was performed with school children and different HRQoL aspects with different measurements were used.

The study did not reveal any significant relationships between types of performance-based sports (individual- and team-based sports) and other HRQoL dimensions, including psychological well-being, social support and peers and school environment. It could be that there was a grey area and less clear distinction between individual- and team-based sports, which made it difficult to establish the association [20]. Therefore, individual and team sports seem to have equal advantages for psychological well-being and social support & peers, in line with a previous study carried out among children [20]. Team sports provide opportunities to build social relationships and a sense of belonging [22, 75]. It provides not only a chance to be physically active but also sociable, such as making new friends, interacting with coaches, and building teamwork [76]. However, youths in individual sports also often train in group settings, similar to their peers in team sports, and therefore are likely to experience comparable group dynamics and social processes [77]. Teenagers in individual-based sports could still be sociable, for example with peers and the coach during practice and have friendships with peers during other activities. Especially when presence of a friend was previously reported to be significantly associated with youth’s motivation to be physically active [78]. Adolescents are sensitive to pressure from peers and need to please significant adult figures, they may experience pressure to stay fit, improve their performance, strength, endurance and muscle [66]. A possible common goal between peers in individual-based sports may exist. Similarly, teenagers in team sports could engage in mutual helping, enhance the interpersonal liking and harmony with the same team members through common goal, outcome interdependence and resource interdependence [18, 79, 80].

We have found that adolescents who participated in individual-based sports had worse parent relations and autonomy than adolescents who did not participate in sports. Supporting literature about our findings is scarce. The non-athlete group had a better perception of their autonomy and relatedness with their parents than the individual athlete group. It could probably be explained by worse secure attachment experienced by individual athlete adolescents, as parents from sport inactive adolescents were previously reported to perceive significantly less time pressure than parents of adolescents that participated in only individual sport [81]. As part of the demands of having a child engaged in sport, parents may feel life stress and time pressure in regards for arranging the organisational matters e.g., managing the child injury, arranging transportation, covering the finacial cost, or even sport activity preparation [81–84]. As such, it collides with the parents’ ongoing schedule and often affects time for their own physical and mental health and sometimes decreases time spent with family [81, 85]. This could probably explain why teenagers in individual sports have a subjective perspective of lacking parents’ availability to talk and spend time together. Different from young children who require physical availability and close proximity, adolescents may have secure attachment and feel comfort by recognizing that their parents are sympathetic despite their absence [86]. Furthermore, parental attunement and sensitivity remain important in the parent attachment, especially in autonomy aspect [86]. It may be that adolescents in individual sports group received pressure from parents that might lead them to have less free will and autonomy. A study showed that adolescents not participating in sport had worse parent attachment than adolescent athletes [87]. It could be that the relationship with parents among individual sport groups might lead to parents’ encouragement, which has been found to be important in developing sport involvement in youth athletics [88, 89]. However, parents’ encouragement sometimes could also be pressure, causing anxiety and burnout through the inducement of perfectionistic expectations [88, 90, 91]. It is important to note that the study was performed among athletes aged 12–16 years old and general competitive sport were reported. Competitiveness level and training intensity of the sports, in which the adolescents were involved, were not investigated in the current study and should be further studied in future study.

### Strengths and limitations

A significant relationship was found between sports participation and physical well-being in the current study. Different performance-based sport types, particularly individual- and team-based sports and their association with physical well-being and parent relations and autonomy were explored. Analysis included the adjustment for subjective SES and migration background. To our knowledge, no prior study has investigated the associations between sports participation as well as various performance-based sport types and HRQoL in adolescents. The present study fills this gap and provides a significant contribution to the literature on sports and quality of life in Germany. This study could offer valuable insights and provide a better understanding for future analysis and elaboration in development of sport programs among adolescents. In addition to relevant findings on HRQoL in adolescents, our study utilized self-reported measurements rather than parent-reported data. In a healthy population, as in our case, this could provide a better indication of the HRQoL for teenagers than what parents reported [92, 93].

Several limitations, however, should be considered. The potential bidirectional effects and causal relationships between participation in sports or performance-based sports types and HRQoL could not be determined with a cross-sectional design [20, 94, 95]. Only statistical associations could be determined. Furthermore, our sample comprises only adolescents from one city in Germany, and it will be difficult to generalize the results at national and international levels. Furthermore, findings from the multivariate analysis should be interpreted with caution because the dependent variable HRQoL was not normally distributed. A future study with a larger sample from different cities in Germany is suggested. Further research that considers other sports, related factors-such as intensity, competitiveness, and training environment, is essential for a more comprehensive understanding of the impact of sports participation on adolescents’ HRQoL. Furthermore, such study could be advantageous for providing robust scientific evidence supporting guidelines for adolescent health programs.

## Conclusion

Our findings suggest that being active in sports is linked to better physical well-being than being sport inactive among adolescents. Whether it is individual, team, or both individual and team sports, they appeared to be associated with favorable physical wellbeing. However when considering individual and team sports altogether, adolescents showed the most favorable physical wellbeing. Our sample included adolescents from only one city in Germany, limiting generalizability of the findings. Adolescents should participate and engage in any sports to maintain their physical health. Participating in individual and teams sports outside school followed by physical education in schools could support the adolescents to be physically active and may improve the likelihood to meet physical activity recommendation. Organised sports participation, which declines from children to youth, could be good option as leisure time activities and could be part of adolescents’ daily life to spend day better in the modern society. Furthermore, individual and team sports could be valued places for adolescents as it could facilitate positive development. In this case, parents play an important role to support the health and development of their children by providing positive encouragement and parental involvement. The facilitation of various individual and team sports to reduce barriers of sports participation for adolescents may be a prominent aspect of promoting adolescents’ health. Other factors related to sports such as competitiveness and intensity level could be incorporated in the future studies to gain a better understanding of sports participation and HRQoL among adolescents.

## Figures and Tables

**Figure 1:**
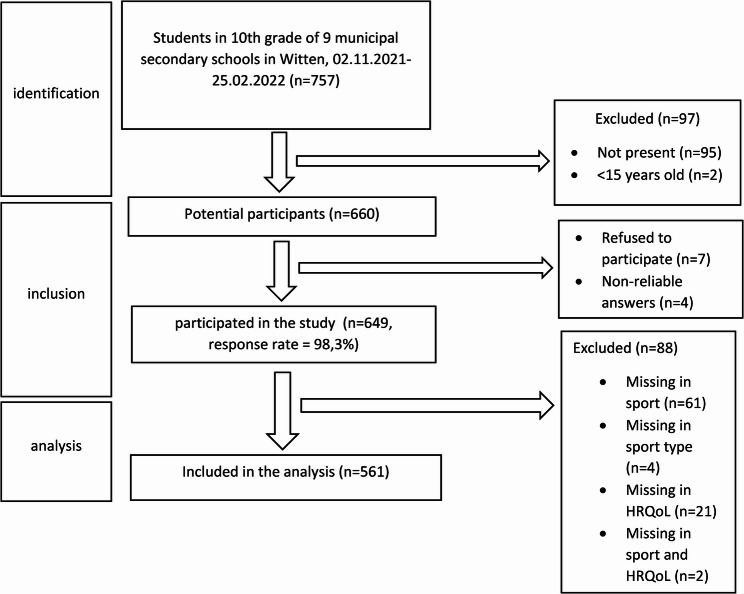
Flowchart of GeWIT-Study Participants Inclusion

## Data Availability

The datasets generated and/or analyzed during the current study are not publicly available due to data protection but are available from the corresponding author upon reasonable request.

## Appendix 2

**Table 4:** Sports Names Identified by Participants and Performance-Based Groupings Derived from Their Responses

Individual-based sports^a^	Team-based sports^b^	Individual- and team-based sports
Athletics	American Football	Rowing
Badminton	Baseball	Kayak
Ballet	Basketball	
Bodybuilding	Dragon boat	
Bowling	Football	
Boxing	Handball	
Circus	Hockey	
Climbing	Irish dance	
Cycling	Pair dancing	
Dancing	Volleyball	
Equestrian vaulting		
Fighting sport		
Fitness training		
Golf		
Gymnastics		
Hiking		
Home workouts		
Horse riding		
Hulla-hoop		
Ice skating		
Jogging		
Judo		
Karate		
Kenjutsu		
Motorcross		
Parkour		
Skateboarding		
skating		
Swimming		
Sword fighting		
Table Tennis		
Tennis		
Windsurfing		
Yoga		

^a^[23, 26, 30, 31, 96]

^b^[22, 23, 30, 31, 96]

## Abbreviations

BMI: Body Mass Index
GeWIT Study: Gesunde Stadt Witten Studie (Healthy City Witten Study)
HBSC: Health Behavior in School Age Children
HRQoL: Health-Related Quality of Life
iamag: Institut für Allgemeinmedizin und Ambulante Gesundheitsversorgung or Institute of General Practice and Primary Care
Kg: Kilogram
KiGGS study: German Health Interview and Examination Survey for Children and Adolescents
M: Meter
MVPA: Moderate to Vigorous Physical Activity
RKI: Robert Koch Institute
SD: Standard Deviation
SES: Socioeconomic Status
WHO: World Health Organization
